# Analysis of Biotinylated Generation 4 Poly(amidoamine) (PAMAM) Dendrimer Distribution in the Rat Brain and Toxicity in a Cellular Model of the Blood-Brain Barrier

**DOI:** 10.3390/molecules180911537

**Published:** 2013-09-17

**Authors:** Ruth Hemmer, Andrew Hall, Robert Spaulding, Brett Rossow, Michael Hester, Megan Caroway, Anthony Haskamp, Steven Wall, Heather A. Bullen, Celeste Morris, Kristi L. Haik

**Affiliations:** 1 Department of Biological Sciences, Northern Kentucky University, SC 204, Highland Heights, KY 41099, USA; E-Mails: hemmerr1@nku.edu (R.H.); halla19@nku.edu (A.H.); rtspau01@louisville.edu (R.S.); bmross03@louisville.edu (B.R.); hesterm2@gmail.com (M.H.); carowaym1@gmail.com (M.C.); 2 Department of Chemistry, Northern Kentucky University, SC 450, Highland Heights, KY 41099, USA; E-Mails: haskampa1@gmail.com (A.H.); walls1@nku.edu (S.W.); 3 Center for Integrative Natural Science and Mathematics (CINSAM), Northern Kentucky University, FH 519, Highland Heights, KY 41099, USA

**Keywords:** dendrimers, atomic force microscopy, fluorescence microscopy, biotin, toxicity, blood brain barrier, lactate dehydrogenase assay

## Abstract

Dendrimers are highly customizable nanopolymers with qualities that make them ideal for drug delivery. The high binding affinity of biotin/avidin provides a useful approach to fluorescently label synthesized dendrimer-conjugates in cells and tissues. In addition, biotin may facilitate delivery of dendrimers through the blood-brain barrier (BBB) via carrier-mediated endocytosis. The purpose of this research was to: (1) measure toxicity using lactate dehydrogenase (LDH) assays of generation (G)4 biotinylated and non-biotinylated poly(amidoamine) (PAMAM) dendrimers in a co-culture model of the BBB, (2) determine distribution of dendrimers in the rat brain, kidney, and liver following systemic administration of dendrimers, and (3) conduct atomic force microscopy (AFM) on rat brain sections following systemic administration of dendrimers. LDH measurements showed that biotinylated dendrimers were toxic to cell co-culture after 48 h of treatment. Distribution studies showed evidence of biotinylated and non-biotinylated PAMAM dendrimers in brain. AFM studies showed evidence of dendrimers only in brain tissue of treated rats. These results indicate that biotinylation does not decrease toxicity associated with PAMAM dendrimers and that biotinylated PAMAM dendrimers distribute in the brain. Furthermore, this article provides evidence of nanoparticles in brain tissue following systemic administration of nanoparticles supported by both fluorescence microscopy and AFM.

## 1. Introduction

In recent years, the field of nanotechnology has emerged as an important area of biomedical research. Nanoparticles (NPs) are studied in a variety of biological systems, and the use of NPs as novel therapeutic agents has been described in several experimental systems, such as cancer, eye diseases, diabetes, coronary artery disease, *etc.* [1,2,3,4]. While this disease model has shown promise for the use of NPs and nanocarrier drug delivery systems, the issues of biodistribution and toxicity need to be addressed. The nanosize dimensions of NPs have been reported to facilitate the crossing of several biological barriers such as the skin, tight junctions of various epithelial layers, and the blood-brain barrier (BBB) [5,6].

The BBB is a tight barrier of cells which separates the circulating blood from the central nervous system (CNS). The walls of BBB capillaries are composed of brain capillary endothelial cells (BCEC), which form tight junctions. Tight junctions contain integral membrane proteins that form a seal between adjacent endothelial cells. In addition, accessory structures that surround the BCECs include pericytes, associated astrocytes and neurons [7,8,9]. While the BBB is essential for maintaining CNS function and homeostasis, it is also a major obstacle in the treatment of many brain diseases. The poor permeability of various drugs and delivery systems across the BBB is primarily due to tight junctions, lack of capillary fenestrations and presence of efflux transporters. The BBB can reportedly block more than 98% of CNS drugs [10]. Consequently, finding new ways to deliver therapeutic drugs to the CNS safely and effectively is essential.

Various drug delivery and targeting strategies to overcome the BBB are under investigation, and a number of nanoparticle delivery systems have shown promise [10,11,12,13]. One approach is the use of surface-modified polymeric nanoparticles as drug carriers, such as dendrimers. Dendrimers are an appealing choice for nanoparticle drug delivery because of their highly branched and complex architecture, uniform size, internal cavities, high loading capacity, low toxicity and low immunogenicity [14,15,16,17]. The presence of a large number of surface groups provides opportunity to conjugate ligands not only for transport across the BBB but also for targeting to specific cells, such as tumors. Dendrimers can be prepared with specific surface modifications that enable the dendrimers to gain entry through a membrane while holding a molecule that cannot pass on its own. Once the dendrimer passes the membrane, it may serve as a therapeutic transporter.

Due to dendrimer versatility, there are a tremendous number of potential applications for dendrimers in nanomedicine with poly(amidoamine) PAMAM dendrimers being the most extensively studied [16,18,19,20,21,22,23]. Several targeted drug delivery systems utilizing various targeting ligands have been used with some success in terms of BBB crossing [10,13] including lactoferrin [24], epidermal growth factors [25] and doxorubicin [26]. However, the mechanisms of uptake and toxicity to the BBB have not been extensively studied. A detailed characterization of dendrimer biodistribution and toxicity is important for the design and use of dendrimers in brain drug delivery.

Biotin is an important molecule used in several metabolic pathways throughout the body [27,28]. Biotin has been shown to cross the BBB, suggesting that biotinylated PAMAM dendrimers may also have the potential for delivering therapeutic drugs to the brain [27,29,30]. Biotin-labeled dendrimers have been utilized in tumor [31] and antibody targeting [32] studies and biosensor design [33].

Atomic force microscopy (AFM) provides 3D mapping of a surface on the nanoscale, and was utilized as a complementary method to evaluate dendrimer distribution in the dorsal striatum of the rat brain. AFM has been recently applied to reveal PAMAM dendrimers on mica, *in vivo* brain tissue measurements [34,35,36], and subcellular features in rat brain tissues using phase imaging [37,38]. AFM has also been used to evaluate neuron growth [39], β-amyloid fibril aggregation [40,41,42], disruption of microtubulin structures [43], and to measure the mechanical differences between white and gray matter in rat cerebellum [44]. These results provide important insights into strategies for developing nanoparticle systems for brain drug delivery.

In this study, distribution of dendrimers *in vivo* and potential toxicity of biotinylated and non-biotinylated G4 PAMAM dendrimers were evaluated utilizing a lactate dehydrogenase (LDH) toxicity assay, fluorescence microscopy, and AFM. This is the first report of visualizing G4 PAMAM dendrimers in rat brain using AFM.

## 2. Results and Discussion

### 2.1. Toxicity in Co-Culture

While there is evidence of toxicity with PAMAM dendrimer use, strategies have been developed to minimize this toxicity [30,45]. Several surface modifications to decease toxicity of cationic PAMAM dendrimers have been attempted, including substitution of surface amine groups with polyethylene glycol [46], acetyl groups [47], and hydroxyl groups [45]. In this study, G4 PAMAM dendrimers were conjugated to biotin prior to evaluation of toxicity. Because biotin is found throughout the body, we hypothesize that dendrimers functionalized with biotin would be non-toxic. 

Biotin has been shown to cross the BBB through carrier-mediated endocytosis that does not metabolize the biotin molecule [48]. Given the ubiquitous nature of biotin *in vivo*, it is possible that biotinylated PAMAM dendrimers could potentially cross the BBB via carrier-mediated endocytosis without compromising the BBB.

A co-culture model of the BBB consisting of primary astrocytes and primary endothelial cells was chosen for *in vitro* toxicity analysis based on previous research outlining which cell culture models of the BBB are most appropriate for BBB functionality and transport studies [49,50]. To measure cell toxicity, an LDH assay was used. LDH is a cytosolic enzyme that is released into the culture medium after the cell membrane has been compromised. When cell-free aliquots of the medium from cultures given different treatments are assayed, the amount of LDH activity can be used as an indicator of relative cell viability. The LDH assay has been used extensively as an alternative to ^51^Cr release for cell mediated cytotoxicity assays. Additionally, this assay has been used successfully to evaluate NP toxicity in several *in vitro* systems [51,52]. In this experiment, cell viability was measured spectrophotometrically at 24 and 48 h time intervals. An observed increase in absorbance directly correlates to the level of cellular damage to the co-culture model.

**Figure 1:**
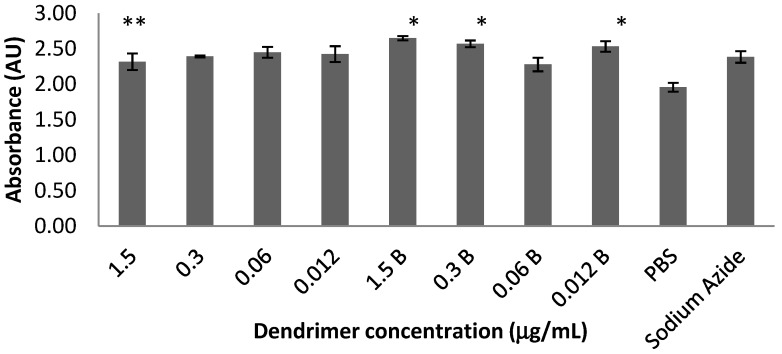
Biotinylated G4 PAMAM dendrimers induce toxicity in the *in vitro* BBB model at the 48 h time point. There was no notable difference between the non-biotinylated dendrimers and cells treated with phosphate buffered saline (PBS; negative control) or sodium azide (positive control). Ryan’s (R-E-G-W-Q) *post-hoc* analyses revealed the following differences: * *p* < 0.05 when compared to PBS controls; ** *p* < 0.05 when compared to 1.5 μg/mL biotinylated dendrimers. All of the biotinylated dendrimers except 0.06 µg/mL were toxic when compared to PBS, and none differed from sodium azide, indicating cell toxicity. Bars 1–4 represent non-biotinylated dendrimers and bars 5–8 represent biotinylated dendrimers. Values represent means ± SEM.

Repeated-measures analysis of variance (ANOVA) showed a significant difference between 24 and 48 h time points, [F(1,16) = 391.798, *p* < 0.001]. However, no significant differences appeared when both time periods were analyzed simultaneously. The data from the 48 h time point were then isolated and analyzed separately and are shown in Figure 1. A one-way ANOVA was used to compare dendrimer dose combinations with the PBS and sodium azide controls followed by a *post-hoc* Ryan’s test (*i.e.*, R-E-G-W-Q), [F(9,20) = 4.370, *p* < 0.01]. All concentrations of the biotinylated dendrimers except 0.06 µg/mL showed elevated LDH compared to PBS, while none differed from sodium azide (positive control for toxicity).

These results indicate that both G4 biotinylated and non-biotinylated PAMAM dendrimers were shown to be non-toxic to a co-culture cellular model of the BBB at the 24 h time point (data not shown). However, after 48 h of exposure the co-culture showed significant toxicity with the biotinylated PAMAM dendrimers compared to the non-biotinylated PAMAM dendrimers. It is possible that the co-cultures exposed to biotinylated dendrimers show significant toxicity differences after 48 h as a result of higher accumulation of dendrimers in cells due to the carrier mediated endocytosis mechanism utilized by biotin to cross the BBB [27,28]. In other words, after 48 h of exposure the biotinylated dendrimers gained access to a greater number of cells in the co-culture compared to the non-biotinylated dendrimers, corresponding to an increase in cell death. The exact mechanism of dendrimer toxicity remains unknown. Factors affecting dendrimer toxicity include size, external functional groups, surfactants as well as drug loaded [53,54], although toxicity due to biotin cannot be ruled out [27,28].

### 2.2. Fluorescence Intensity of Rat Brain, Kidney, and Liver Tissues

**Figure 2:**
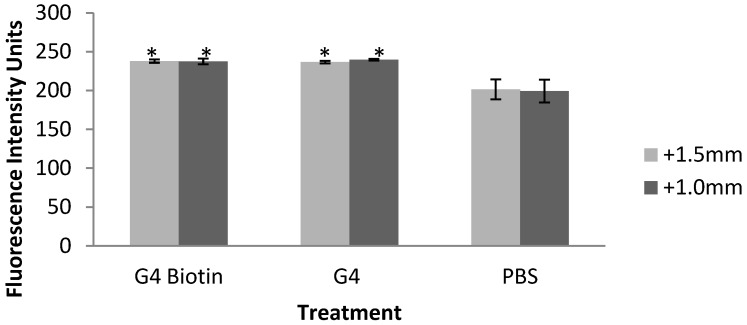
Fluorescence intensity measurements of rat brain dorsal striatum at 2 levels of bregma (+1.5mm and +1.0mm) in rats exposed to G4 PAMAM dendrimers, biotinylated G4 PAMAM dendrimers, and PBS (control). Values represent mean ± SEM. * *p* < 0.05 compared to PBS controls using Tukey *post-hoc* analyses.

Fluorescence intensity measurements in rat brain tissue obtained 24 h after systemic injection of dendrimers indicated that non-biotinylated and biotinylated PAMAM dendrimers were detected in the rat dorsal striatum (*caudate putamen*) [55,56]. The dorsal striatum was chosen for analysis based on evidence that this brain region may have a weakened BBB following neurotoxic insult. Rodents injected systemically with 3-nitroproprionic acid show lesions in the dorsal striatum induced by this neurotoxin. It is hypothesized that neurotoxin-induced increases in BBB permeability in the dorsal striatum is linked to damage of vascular endothelial cells and laminin [57,58]. Although we cannot determine whether the NPs crossed the BBB, one possibility is that the presence of dendrimers in the dorsal striatum indicates an alteration in BBB permeability. ANOVA on fluorescence intensity measurements in brain indicate that dendrimers may deposit in the dorsal striatum of G4 and G4 biotinylated-treated rats (Figure 2) at +1.5 from bregma, [F(2,5) = 12.846, *p* < 0.05], and +1.0 mm from bregma, [F(2,5) = 11.225, *p* < 0.05]. Tukey *post-hoc* analysis shows that tissue treated with both types of dendrimers were significantly different than PBS-treated tissue.

While both dendrimers may accumulate in the dorsal striatum, the conjugation of the avidin fluorophore would only occur with the biotinylated dendrimers, based on the affinity of avidin and biotin [31,33].The increase in fluorescence intensity of non-biotinylated dendrimers may be based on an autofluorescence property of PAMAM dendrimers [55]. 

Distribution of PAMAM dendrimers in brain tissue varies depending on the surfactant and model. Using PEG-PAMAM-Doxorubicin dendrimers in a murine model of glioma, Zhang *et al.* demonstrated dendrimer accumulation in the brains of mice, and the accumulation was higher in brain tumor than normal tissue [1]. In another report, the delivery method played a role in brain accumulation of dendrimers. PAMAM dendrimers complexed with sulfadiazine showed greater accumulation in the brains of rats following intravenous administration compared to oral administration [59]. Dai *et al.* showed that PAMAM dendrimers conjugated to N-acetyl cysteine localized to specific brain cells, activated microglia and astrocytes, in newborn rabbits with maternal inflammation-induced cerebral palsy [60]. In contrast, PAMAM dendrimers, both uncoupled and coupled to PEG, showed no accumulation in the brain tissue of KB tumor cell-bearing nude mice 6 h after intravenous injection [61,62]. More research is needed to fully understand the localization, activity and impact on the BBB of PAMAM dendrimers in the brain.

Data from the kidney [F(2,4) = 3.701, *p* > 0.05] and liver [F(2,5) = 2.092, *p* > 0.05] indicate that there were no significant differences between treated and non-treated rats. The lack of significance in these tissues may be because accumulation of dendrimers in organ tissue was lower than the signal-to-noise fluorescent intensity of the instrument. One possible explanation for the similarities in fluorescence intensity in dendrimer-treated and control kidney and liver tissues is that endogenous biotin is masking the presence of biotinylated dendrimers due to non-specific avidin-biotin binding. In has been shown in rats that high levels of endogenous biotin are present in both kidney and liver [63,64,65].

However, in the rat brain, biotin has been detected in several areas, including the substantia nigra of the basal ganglia but not in the dorsal striatum (the area analyzed in this study) or anywhere else in the *caudate nucleus* or *putamen* [66,67]. This lack of endogenous biotin in the rat brain dorsal striatum supports our findings of increased fluorescence intensity of treated and non-treated brain tissue. We suggest that no difference was observed between biotinylated and non-biotinylated dendrimers due to the autofluorescence property of PAMAM dendrimers [55].

### 2.3. AFM Analysis

**Figure 3:**
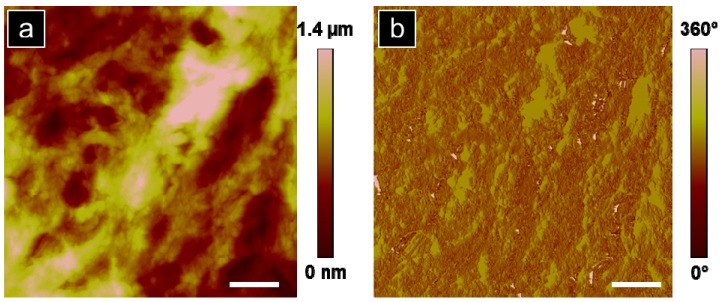
Representative AFM analysis of the dorsal striatum (30 × 30 µm) from a rat treated with G4 biotinylated PAMAM dendrimers: (**a**) topographic and (**b**) phase images. Scale bar equal 5 µm.

Atomic force microscopy of the rat dorsal striatum post systemic injection of G4 biotinylated PAMAM dendrimers was performed to provide physical evidence of the presence of dendrimers in brain and to further suggest that the detected fluorescence signal was due to the presence of dendrimers in the brain following systemic injection. A representative topographic image of a rat dorsal striatum treated with G4 biotinylated PAMAM dendrimers obtained 24 h after systemic treatment is shown in Figure 3a with the corresponding phase image in Figure 3b.

**Figure 4:**
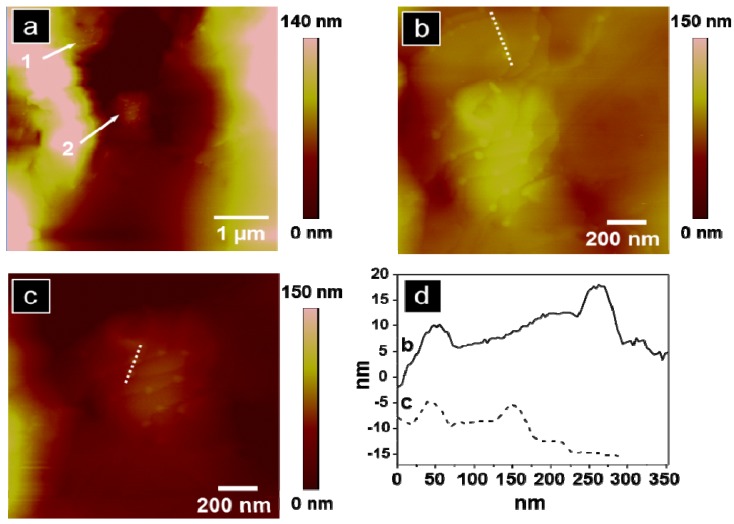
Topographic AFM images of the dorsal striatum from a rat treated with G4 biotinylated PAMAM dendrimers: (**a**) topography of brain capillary with two regions of interest marked; (**b**) zoomed-in analysis (1.4 × 1.4 µm) of region 1 from (**a**); (**c**) zoomed-in analysis (1.4 × 1.4 µm) of region 2 from (**a**); (**d**) representative cross-sectional analysis for (**b** and **c**).

The undulating features reveal variation in topography of 1.4 µm. A more detailed topographic analysis in a clearly depressed region of tissue, significant of a brain capillary, shows evidence of dendrimers in the inner capillary wall and demonstrates PAMAM dendrimers present at the BBB, Figure 4a, regions (1) and (2). Figure 4b,c are zoomed-in images of regions (1) and (2) from Figure 4a, respectively. Cross-sectional line scans along the dotted segments in Figure 4b,c are illustrated in Figure 4d. The particle heights obtained from four distinct particles represented in Figure 4d range from 2.7–10 nm which agrees with dendrimer size obtained by AFM analysis reported by Li *et al.* and with dendrimer analysis observed in our previous work [38,68]. Feature width, from Figure 4d, is 30–50 nm which is 7–13 times larger than the expected diameter of G4 PAMAM dendrimers, 4 nm [69]. While the topographic height measurements obtained by AFM approximates the G4 PAMAM dendrimer size (Figure 4d), the particle width overestimates the true G4 PAMAM dendrimer diameter. We propose that the measured particles could be aggregates of dendrimers which contribute to the overestimated width obtained from the cross-sectional line scan. Additionally, convolution is innate with scanning probe measurements due to probe-surface interactions, especially present with highly convoluted, soft features characteristic of tissue samples [70]. Topographic AFM images point to the presence of dendrimers within a capillary, but not further in surrounding tissue, Figure 4a. Particle location observed with AFM augments the importance of incubation time as a factor in particle penetration depth in brain tissue. Future studies will increase time and frequency of exposures to dendrimers to determine if dendrimers remain in brain capillary or cross the BBB into brain parenchyma.

**Figure 5:**
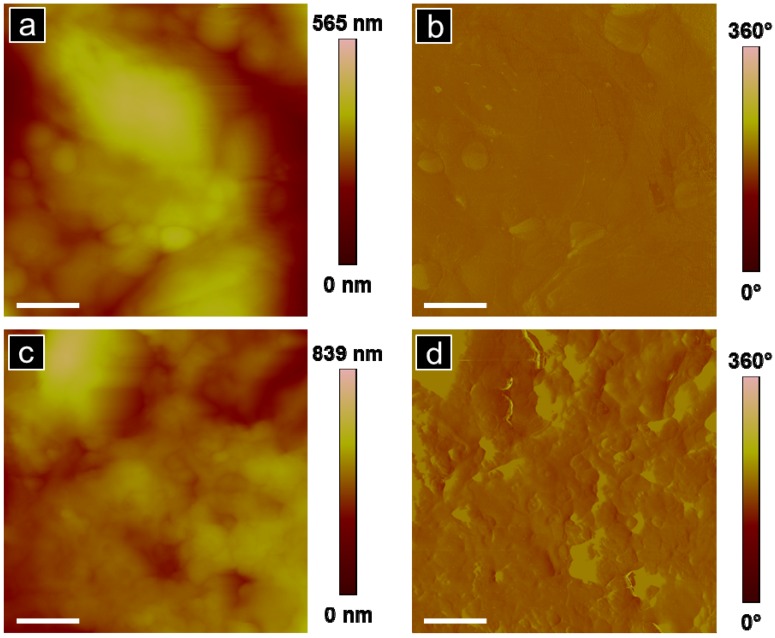
(**a** and **b**) AFM analysis of the dorsal striatum of a rat treated with G4 biotinylated PAMAM dendrimers: (**a**) topography and (**b**) phase image in a region where no dendrimers are observed. (**c** and **d**) AFM analysis of an untreated rat dorsal striatum (no systemic treatment of G4 biotinylated PAMAM dendrimers): (**c**) topography and (**d**) phase image. Scale bar is 1 µm for all images.

While evidence of biotinylated G4 PAMAM dendrimers was observed in rat brain tissue, we also observed regions of the striatum without evidence of dendrimers taken from the same sample, Figure 5a,b. Regions of brain tissue without dendrimers, even in brains obtained from systemically treated rats 24 h post incubation, were reproducibly observed, *n* = 3. To further support that particles observed in the brain section corresponded to G4 biotinylated PAMAM dendrimers, an untreated rat brain was imaged with AFM, Figure 5c,d. The tissue region from dendrimer-treated rat brain (Figure 5a) and the untreated rat brain (Figure 5c) display similar nanoscale features of tissue but are in contrast to the image at the same magnification of treated rat brain with small particle packets clearly observed in Figure 4a.

Thus, brain tissue from rats which had been systemically treated with biotinylated G4 PAMAM dendrimers demonstrated both regions containing packets of particles indicative of dendrimers and regions without such features which could correspond to dendrimer-tissue penetration related to incubation time.

## 3. Experimental

### 3.1. Dendrimer Preparation

Poly(amidoamine) PAMAM dendrimers [core: ethylene diamine]; (G = 4); *dendri-*PAMAM-(NH_2_)_32_) were obtained from Dendritic Nanotechnologies, Inc. (Mt. Pleasant, MI, USA). Biotinylated PAMAMs were prepared using sulfo-NHS-LC-biotin (Pierce EZ-Link^®^ Kit) as described previously [71]. Biotinylated dendrimers had approximately 17% functionalization which corresponds to 11 surface groups per dendrimer, as shown by nuclear magnetic resonance (NMR) spectroscopy [68]. Biotinylated dendrimers were resuspended at 1.0 mg/mL in 0.1 M PBS (pH 7.4) at 4 °C until used. All organic solvents used were analytical, HPLC grade, from Sigma (Sigma-Aldrich; St. Louis, MO, USA). DI water was obtained using a Milli-Q plus water purification system (Millipore; Bedford, MA, USA). PBS and Borate buffers were prepared from Pierce buffer packs (Pierce Protein Research Products; Rockford, IL, USA).

### 3.2. Cell Co-Culture

Astrocytes and BCEC were isolated from rat brain cortex and cultured using procedures adapted from Garcia-Garcia *et al.* and Szabó *et al.* [72,73]. To confirm the purity of the astrocyte cultures immunocytochemical staining with an antibody to glial fibrillary acidic protein (DAKO; Carpinteria, CA, USA) was performed; likewise the purity of the BCEC cultures was confirmed via immunocytochemical staining with an antibody to rat endothelial cell antigen-1 (AbD Serotec; Raleigh, NC, USA) (data not shown). Culture media for astrocytes was composed of DMEM/F12 containing 20% FBS and an antibiotic solution of 10,000 units/mL of penicillin, 10,000 µg/mL of streptomycin, and 25 µg/mL of Fungizone^®^ (Life Technologies; Grand Island, NY). Culture media for BCEC was composed of EBM^TM^-2 culture medium supplemented with EGM^TM^-2 MV Singlequots^®^ (Lonza; Walkersville, MD, USA). Early passage astrocytes and BCEC were used for co-culture experiments. The co-culture media was 1:1 astrocyte media and BCEC media. Astrocytes were plated on a 96-well plate at 30,000 cells/well and 24 h later BCEC were plated at 30,000 cells/well on top of the astrocytes. The cells were grown together for 6 days and then exposed to G4 and G4-biotinylated PAMAM dendrimers (three wells/condition) at the following concentrations: 1.5 µg/mL, 0.3 µg/mL, 0.06 µg/mL, 0.012 µg/mL. The dendrimers were diluted in 0.1 M PBS at pH 7.4. PBS served as a negative control. Treatment of cells with 1% sodium azide was performed as a positive control for toxicity.

### 3.3. Lactate Dehydrogenase (LDH) Toxicity Assay

Cell viability was determined using the LDH release method in the TOX-7 LDH based *in vitro* toxicology assay kit (Sigma). The assay was performed on media collected 24 and 48 h after exposure to G4 and G4-biotinylated PAMAM dendrimers. LDH reduces NAD+, which converts a tetrazolium dye to a colored formazan derivative, that is detectable at a wavelength of 490 nm and subtracted from a background of 690 nm [74,75,76]. A repeated-measures ANOVA was used to compare all doses over 24 and 48 h time points. Separate two-way ANOVAs were used to compare all treatment doses of both dendrimer types at 24 h and then at 48 h. Finally, separate one-way ANOVAs were used to compare non-biotinylated dendrimers, biotinylated dendrimers and controls. ANOVAs were followed by Ryan’s *post-hoc* analysis (R-E-G-W-Q) when appropriate (IBM SPSS Statistics 19, IBM; Armonk, NY, USA).

### 3.4. Animals

All rats were treated in accordance with the National Institutes of Health PHS Policy on Humane Care and Use of Laboratory Animals (2002) and the Northern Kentucky University Institutional Animal Care and Use Committee (IACUC). All animal protocols were reviewed and approved by the Northern Kentucky University Animal Care and Use Committee. Eight male Sprague-Dawley^®^ rats (Harlan Laboratories; Indianapolis, IN, USA) were house in large Plexiglas cages and maintained on a 12:12 h light:dark cycle. Food and water were available *ad libitum* throughout the experiment.

### 3.5. Tail Vein Injections

Six month old Sprague-Dawley rats were anesthetized with 60 mg/mL sodium pentobarbital (1 mL/kg body mass) and their tails were dipped into warm water prior to injection to dilate the tail vein. Animals (weight 0.362 kg ± 0.074 kg) were injected with either 0.5 mL of 0.1M PBS, 0.5 mL of G4 PAMAM dendrimer (500 nM), or 0.5 mL of biotinylated G4 PAMAM dendrimer (500 nM).

### 3.6. Brain Tissue Preparation

Twenty-four hours after tail vein injections, rats were anesthetized with 1.0 mL of sodium pentobarbital (60 mg/mL). Rats were then perfused with 50 mL of 0.1M PBS followed immediately by 200 mL of 4% paraformaldehyde made in 0.1 M phosphate buffer (PB). Brain, kidney, and liver tissues were removed, placed in 30% sucrose for cryopreservation until they sank, and then shock frozen with dry ice. All tissues were sectioned at 60 µm on a cryostat at −20 °C and placed in PBS with 0.05% sodium azide (Sigma, to inhibit fungal growth). Brain slices used for fluorescence intensity and AFM of the dorsal striatum were taken at +1.5 and +1.0 mm from bregma. The center point of the sampling region for both sections is medial/lateral (ML) ±2.2 mm and dorsal/ventral (DV) −3.2 mm [77].

### 3.7. Fluorescence Intensity in Rat Brain, Kidney and Liver Tissues

Two brain slices per level of bregma (+1.5 and +1.0 mm; described in 3.6) were incubated for 1 h in 4 mg/mL AlexaFluor^®^488-Avidin in PBS (Life Technologies) [77] in a dark room. Following staining, sections were rinsed three times in PBS and mounted onto gelatin-coated microscope slides and coverslipped with Aqua Poly/Mount mounting media (Polysciences, Inc.; Warrington, PA, USA). Stained specimens were imaged with a Nikon Eclipse E600 confocal microscope equipped with a Nikon 40× objective lens. The emission wavelength was 488 to detect the AlexaFluor^®^488-Avidin marker. Gains were set and maintained constant for all specimens and all specimens were imaged in the same session. Acquired images were run through Metamorph (v6.1) software (Diagnostic Instruments, Inc., Sterling Heights, MI, USA) for fluorescence intensity measurements. An area of tissue was selected using regional tools to ensure sizes were identical. Regional measurements were taken and “average intensity” was selected as the dependent variable [56]. Additional controls were measured to determine scale (e.g., no light control and white light control).

### 3.8. AFM Analysis of Brain Tissue

A Dimension 3100 Digital Instruments SPM was utilized in tapping mode (NSC 14 tip- 140 kHz) for all image analysis. Typical scan rates were 1 Hz and all images were acquired in air. Brain tissues were removed from PBS and 0.05% sodium azide solution and placed on glass microscope slides for analysis. The AFM images presented were collected in the dorsal striatum (identical to the area for fluorescence intensity).

## 4. Conclusions

PAMAM dendrimers are candidates for numerous applications in nanomedicine, yet information regarding their mechanisms of uptake, toxicity and biodistribution is incomplete. We report toxicity of biotinylated and non-biotinylated G4 PAMAM dendrimers in an *in vitro* model of the BBB and we observed that biotinylated dendrimers demonstrate greater toxicity than non-biotinylated dendrimers at 48 h after treatment. Fluorescence microscopy studies on brain tissue obtained 24 h following systemic treatment of dendrimers verified the presence of dendrimers in the dorsal striatum as demonstrated by the increase in fluorescence intensity for treated brain as compared to non-treated brain. Although the data cannot confirm dendrimer crossing of the BBB, we hypothesize that the presence of PAMAM dendrimers in the dorsal striatum indicates an alteration in BBB permeability. To support this analysis, AFM was performed on adjacent brain sections to those used for fluorescence intensity measures. We observed dendrimers in the inner capillary wall, signifying the presence of PAMAM dendrimers at the BBB. These results are significant because they provide both *in vitro* and *in vivo* models for analysis of therapeutic delivery agents. This includes a method to observe NP tissue distribution with both fluorescence microscopy and through topographic images with AFM. Brain tissue analysis with AFM is particularly exciting, due to the high resolution and the capability to observe the nanoscale features of brain tissue and PAMAM dendrimers. Future work includes toxicity, fluorescence microscopy, and AFM analysis of varying PAMAM dendrimer dosages and tissue collection at longer time points after systemic administration, coupled with penetration depth studies to observe relationships between treatment time and dispersion properties of biotinylated G4 PAMAM dendrimers.
